# Addressing Psychosocial Needs in the Aftermath of the Tsunami

**DOI:** 10.1371/journal.pmed.0020179

**Published:** 2005-06-28

**Authors:** Kaz de Jong, Sue Prosser, Nathan Ford

## Abstract

MSF discusses its response to tackling mental health problems in Aceh, Indonesia, and explores some of the main concerns in responding effectively to mental health problems in an emergency setting.

## Teaser

MSF shares its observations from its programme in Aceh, Indonesia

**Figure:**
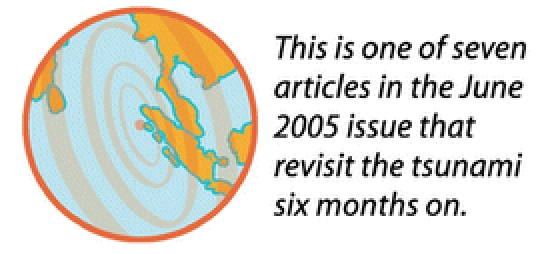


On the morning of 26 December 2004, an earthquake of 9.2 on the Richter scale rocked the province of Aceh, Indonesia, followed soon after by a three-wave tsunami that smashed into the north, west, and topmost point of Aceh. The most heavily hit city, in terms of population, was Banda Aceh on the northernmost point of the Aceh peninsula, exposed between the north and west coast—two-thirds of the city was totally destroyed.

**Figure 1:**
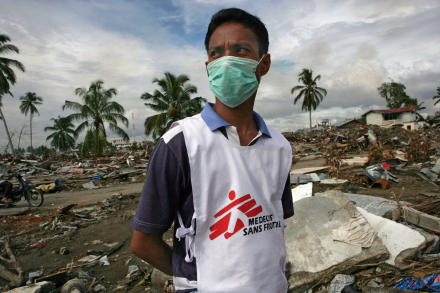
MSF Provides Emergency Medical Support in the Banda Aceh Province of Indonesia In this province, MSF has five areas where teams are actively providing emergency medical support. Here in Meulaboh, 250 km west of Banda Aceh, MSF has started work in the local hospital as well as visiting the surrounding area with mobile teams. Half of the town was destroyed, and access to the area has been by helicopter or boat only. (Photo: © Francesco Zizola/MSF)

Médecins Sans Frontières (MSF) sent four teams consisting of medical, logistical, water and sanitation, and mental health workers (Figure 1), who undertook rapid assessments of the tsunami-affected areas in Aceh Province. This article outlines MSF's response to tackling mental health problems in the region, and discusses some of the main concerns in responding effectively to mental health problems in an emergency setting.

## Assessing Mental Health Needs in an Emergency Setting

Aceh Province in Indonesia has been an area of conflict for more than 40 years. The GAM (Free Aceh Movement) was created in 1959 to pressure the government for independence, and there has been ongoing conflict between GAM and the Indonesian military. The general population is often caught in the middle. Civilians have suffered arrests and intimidation, and have been forced to flee their homes; the tsunami exacerbated what for many was already a fragile existence.

While aid organisations continue to build operational knowledge, there remains considerable debate over the extent to which general mental and psychosocial care programmes provided by these organisations are relevant to the local context [1]. These concerns also extend to the ability of aid organisations to assess needs.

Assessments rely on information gathered using quantitative and qualitative methods. Questionnaires such as the Impact of Events Scale [2], The General Health Questionnaire 28 [3], and the Hopkins Symptoms checklist [4] are used to gather objective data on mental and psychosocial needs. The use of these standard questionnaires has considerable limitations. First and foremost, we should be very modest in our expectations as to what extent it is possible to define the experience of living through a massive crisis in terms of a set of symptoms in a simple checklist. Furthermore, questionnaire tools often lack validation for local context, so they have to be interpreted with caution. Validation, while possible, can take several months and significant resources, and must therefore be done alongside the provision of assistance using approaches proven to be effective in other settings [5].

The limitations to gathering valid quantitative data in the short term requires additional qualitative information gathered through focus group discussions, key informant interviews, social mapping, and literature reviews. The aim is to obtain information that will help interpret individuals' subjective perceptions of their experiences, and to identify levels of resilience and past or present positive coping mechanisms. This information is also used to inform programme design. In the absence of valid and reliable quantitative data, agencies often have to use Western benchmarks.

Western research shows that after exposure to a traumatic event, about 20% of people need some kind of professional psychosocial support to deal with stress and related symptoms or problems, and 5% of people can be expected to have serious mental health disorders [6]. These disorders are mostly combinations of generalized anxiety disorders, post-traumatic stress disorder, and depression. Though the recovery environment in Aceh is less favourable due to the ongoing conflict, MSF uses this estimate in planning programme activities.

## The Emergency and Post-Emergency Mental Health Response

Following initial assessments, and at the request of the Indonesian government, MSF established a community-based psychosocial care programme in January 2005. MSF staff work together with psychologists from the Ministry of Health, providing individual and group counselling.

The primary objective of this programme is to enable people to resume normal activities and to encourage active participation in the community. In Aceh, we use group and individual counselling to help those who are overwhelmed by the events or post-disaster circumstances. The reconnection of individuals with their environment runs parallel to community approaches focused on establishing a climate to facilitate this reconnection. Health education activities covering both physical and mental health issues are used to foster self-help and self-control mechanisms.

The programme promotes understanding and acknowledgement of people's problems, and actively involves formal and informal leaders, such as spiritual and village leaders, whose role in the re-establishment of the previously existing care system is crucial. These approaches are in line with the core strategies to respond to mental health in complex emergencies as defined through collaborative engagement of numerous implementing agencies in recent years (see Box 1).

**Box 1. Refining the Response to Mental Health Needs in Humanitarian Crises**

- **The Sphere Project** (http://www.sphereproject.org) was launched in 1997 by a group of humanitarian NGOs and the Red Cross and Red Crescent movement in an effort to improve the quality of assistance provided to people affected by disaster and to enhance accountability. Chapter 5, “Minimum Standards of Health Service,” has a section on noncommunicable diseases, which includes the standard that “people have access to social and mental health services to reduce mental health morbidity, disability, and social problems.”
- **The Psychosocial Working Group** (http://www.forcedmigration.org/psychosocial), established in 2000, is a collaboration between academic institutions and humanitarian agencies committed to the development of best practice in complex emergencies. Through extensive dialogue between organisations and practitioners representing a range of approaches and orientations to psychosocial intervention, the group has worked to develop a series of papers addressing different issues in psychosocial work.
- **The World Health Organization** has been driving efforts toward a consensus on best public-health practice in respect of mental health. An update on the current state-of-play was given at a recent roundtable discussion, published in the WHO Bulletin (http://www.who.int/bulletin/volumes/83/1/en/71.pdf).

There are only about 500 clinical psychologists for all of Indonesia (about one per 420,000 people), and, in general, most Indonesian people do not know what a psychologist is or does. As a result, we found that mental health issues are often understood in terms of psychiatric disorder and are associated with shame. For this reason, it is important to avoid using psychiatric terms and diagnoses. Instead, in our assessments we frame discussions in terms of wanting to understand people's problems and how people are functioning in their environment.

Another important consideration is how people interpreted the tsunami through their local religions and cultural beliefs. In Banda Aceh, the Muslim religion is central to people's culture and traditions, but mixed with this is superstition and magic. Without exception, the people we spoke with during the assessment understood the tsunami as a punishment or a warning from Allah for being “immoral.” Many believe that the earth will continue to shake until all the dead are buried. Religion is an important tool that helps give people in Banda Aceh meaning and helps them accept what has happened. In our psychosocial activities, we incorporate the importance of religion as a coping mechanism, and we have active links with local religious representatives.

In Banda Aceh, we found that most people have a strong desire to move forward and to rebuild their lives. The men, especially, say that they are anxious about earning a living and supporting their family, and having nothing to do brings them to a state of “great sadness and memories.”

We have also found that it is important to link mental health support with practical support in our psychosocial programmes. Such a link may be a break from the traditional Western approach, where health (physical and mental) is usually considered separate from social and spiritual well being. MSF provides some practical support through health, water, and sanitation programmes. As part of the social activities of these programmes, we establish links on the ground with local and international development organisations, whose role it is to provide practical assistance such as micro-credit schemes, housing, and family-tracing services.

Despite our close collaboration with the existing health system, one problem that persists is the limited possibility for referral of severely traumatized patients to specialized services. There are only three psychiatrists in Aceh Province (which has a population of 4.2 million), and the only psychiatric hospital in Aceh was severely damaged and will not be fully functional for some months. While this hospital is being rehabilitated, mental health care falls to district hospitals and primary health-care clinics, which have little experience in dealing with severe mental health problems.

## The Future

The enormous generosity of the public and governments has generated massive humanitarian assistance in tsunami-affected regions, raising concerns about poor coordination, fragmentation and duplication of relief efforts, and inappropriate assistance [7]. Collaboration between agencies and authorities on the ground is essential, but there is also a fine balancing act between working together and being told what to do. This balancing act is crucially important in areas of conflict where humanitarian agencies have to prevent collaboration from becoming co-optation. Many humanitarian emergencies aid agencies, while working together with governments, have also to insist upon their independence.

In the immediate short term community-based clinical services must be reinforced. Our experience clearly shows that people know how to support each other when distressed but have no referral mechanisms when that support is not enough. The self-help and healing capacity of communities needs to be mobilized in conjunction with culturally adapted psychological interventions in which religion is acknowledged as an important coping mechanism. Primary health-care structures are best placed to provide psychosocial services that are appropriate to the local community. Specialist psychiatric support, while not forming the bulk of the response, does need to be available to those who have more severe problems that cannot be managed at the primary-care level.

The evaluation of large humanitarian interventions in emergency settings is difficult as the quickly changing environment hinders appropriate comparisons, but there is a steadily growing body of research evaluating the effects of psychosocial interventions in emergency settings [8–12]. Culturally specific models and instruments to evaluate programs need to be further developed.

In Banda Aceh, many people expressed the hope that with the presence of Westerners the government will not forget them, and that the oppressive ongoing conflict will not resume as before. In other words, the mere presence of Westerners in the region is valued as important to their current sense of security. This underscores an important principle that holds in many other complex emergencies: not all benefits of humanitarian assistance are amenable to evidence-based assessments [13].

## Abbreviation

MSF: Médecins Sans Frontières
